# Schisandrin B attenuates ischemic acute kidney injury in a CTR1-dependent manner by inhibiting cuproptosis

**DOI:** 10.3389/fcell.2026.1993380

**Published:** 2026-10-15

**Authors:** Lirong Lin, Shahong He, Xujia Yang, Luquan Zheng, Wei Tan, Ke Li, Jurong Yang

**Affiliations:** 1 Department of Nephrology, The Third Affiliated Hospital of Chongqing Medical University (FangDa Hospital), Chongqing, China; 2 Guangyuan Lizhou Secondary Specialized School, GuangYuan, Sichuan, China; 3 Core Research Laboratory, The Second Affiliated Hospital, School of Medicine, Xi’an Jiaotong University, Xi’an, China

**Keywords:** acute kidney injury, cuproptosis, oxidative stress, Schisandrin B, SLC31A1/CTR1

## Abstract

**Background:**

Acute kidney injury (AKI) remains a global health challenge with high mortality rates and no targeted pharmacotherapy. Although regulated cell death (RCD) drives renal tubular epithelial cell (RTEC) injury, the involvement of cuproptosis, a copper-triggered RCD characterized by lipoylated protein aggregation and iron-sulfur cluster loss, remains unexplored in acute kidney injury (AKI).

**Methods:**

Integrated single-cell transcriptomics (GSE180420), a human renal biopsy cohort (*n* = 22), ischemia-reperfusion (I/R) mouse models, and hypoxia-reoxygenation (H/R) cellular models were used to characterize cuproptosis. Multi-omics profiling, molecular docking, and CTR1 (encoded by *SLC31A1*)-specific siRNA knockdown were used to explore the mechanism of action of Schisandrin B (SchB).

**Results:**

Single-cell RNA sequencing of murine ischemia–reperfusion (I/R) kidneys revealed that cuproptosis was a predominant RCD modality, accounting for approximately one-third of all regulated cell death events in renal tubular epithelial cells (RTECs). This finding was corroborated in human AKI biopsies and in murine I/R kidneys, where the key cuproptosis proteins ferredoxin 1 (FDX1) and copper transporter 1 (CTR1) were markedly upregulated, accompanied by significant copper accumulation and tubule injury. Under the tested conditions, SchB exhibited protective effects at least comparable to those of the classical copper chelator tetrathiomolybdate (TTM) in attenuating copper-dependent cytotoxicity *in vitro*. SchB-regulated differentially expressed genes were significantly enriched in glutathione metabolism, p53 signaling, cellular senescence, and PI3K-Akt pathways. Molecular docking predicted a direct interaction between SchB and CTR1 (binding energy −5.1 kcal/mol)siRNA-mediated depletion of CTR1 partially abolished the protective effects of SchB against copper-induced RTEC death, indicating that CTR1 may be required, at least in part, for SchB-induced cytoprotection in RTECs.

**Conclusion:**

This study identifies cuproptosis as a predominant and previously unrecognized RCD pathway in ischemic AKI. SchB inhibits cuproptosis in a CTR1-dependent manner. These findings provide a preclinical rationale for further investigation of this natural product as a potential therapeutic candidate for ischemic kidney disease.

## 1 Background

Acute kidney injury (AKI) affects 13.3 million individuals annually worldwide, with a mortality rate exceeding 50% in critically ill patients. (Zarbock et al., 2024; Pérez-Aizpurua et al., 2024). Despite advances in supportive care, no targeted pharmacotherapies are available. Some patients with AKI may develop chronic kidney disease (CKD), kidney failure, or death (Zarbock et al., 2024; Zhao et al., 2026). The pathophysiology of AKI involves complex interactions among hemodynamic alterations, inflammatory cascades, and regulated cell death (RCD) pathways (Lee et al., 2024; Peng et al., 2024; Wang Y. et al., 2024; Hukriede et al., 2022). The pathogenesis of AKI involves multiple interconnected processes, including oxidative stress (Pavlakou et al., 2017; Liu et al., 2025), inflammation (Song et al., 2025; Meng et al., 2025), endothelial damage, microvascular dysfunction (Patschan et al., 2016) and mitochondrial damage (Wu L. et al., 2024), culminating in renal tubular epithelial cells (RTECs). While autophagy (Cui et al., 2020), necrosis (Kers et al., 2016), apoptosis (Havasi and Borkan, 2011), metabolic disorders (Li et al., 2024a), pyroptosis (Tonnus and Linkermann, 2019), ferroptosis have been extensively studied (Borawski and Malyszko, 2020), emerging evidence suggests that additional RCD modalities contribute to tubular epithelial cell (TEC) loss in AKI.

Cuproptosis, a copper-dependent RCD mechanism characterized by the aggregation of lipoylated mitochondrial enzymes (particularly DLAT) and proteotoxic stress, was first identified by (Tsvetkov et al., 2022), with the copper transporter CTR1 (*SLC31A1*) serving as a key therapeutic target (Zhu et al., 2024; Tsui et al., 2024). Copper is indispensable for the activity of several enzymes involved in mitochondrial oxidative metabolism, oxidative stress defense, and biosynthetic regulation (Tsui et al., 2024). Copper homeostasis is tightly regulated under physiological conditions, with ATP7A/ATP7B-mediated excretion maintaining intracellular copper concentrations below cytotoxic thresholds (Petruzzelli et al., 2025). Ischemia-reperfusion injury disrupts this equilibrium, as copper accumulation has been observed in various AKI models (Shi et al., 2024). Copper overload induces oxidative stress by facilitating Fenton reactions, leading to excessive ROS formation and disruption of mitochondrial homeostasis (Tsui et al., 2024; Duan et al., 2026). This copper–oxidative stress nexus suggests that cuproptosis may represent a previously uncharacterized pathway in ischemic AKI. Among TCM-derived bioactive molecules, several natural compounds have demonstrated promising renoprotective activities in experimental AKI and CKD models.

Schisandrin B (SchB), a dibenzocyclooctadiene lignan isolated from *Schisandra chinensis* (Turcz.), Baill exhibits potent antioxidant, anti-inflammatory, and mitochondrial protective properties in renal ischemia-reperfusion injury. Structurally, the potential protective effect of SchB as a CTR1 modulator may be attributed to its hydrophobic dibenzocyclooctadiene scaffold, which is well-suited for insertion into the extracellular domain pocket of CTR1 (Wang et al., 2025; Jiang et al., 2025; Miao et al., 2022; Cai et al., 2025; Miao et al., 2025). This study hypothesized that SchB attenuates AKI by inhibiting cuproptosis and that this effect is CTR1-dependent (Xu et al., 2024; Ye et al., 2026). Multi-scale models, ranging from molecular docking to human tissue analysis, were employed to validate cuproptosis as a therapeutic target and characterize SchB as a potential CTR1-dependent intervention (Xu et al., 2024; Ye et al., 2026).

## 2 Materials and methods

### 2.1 Single-cell RNA-seq analysis

Single-cell integrated analysis of mouse kidney samples from the GSE180420 dataset was performed using the Scanpy package. The raw data were processed to generate gene expression matrices. Cells expressing at least 200 genes and genes detected in at least 3 cells were retained for further analysis. Unsupervised clustering was performed using the Louvain algorithm (https://githubcom/vtraag/louvain-janossy). To classify the regulated cell death modalities, gene sets representing distinct pathways were scored using sc. tl.score_genes, and each cell was assigned to the pathway with the highest score. RTECs were selected for downstream analysis of the modality proportions. The cuproptosis gene set included FDX1, LIAS, LIPT1, DLAT, PDHA1, PDHB, DBT, GCSH, SLC31A1 (CTR1), ATP7A, and ATP7B genes. Reverse deconvolution​ refers to a computational approach to infer the relative contributions of regulated cell death modalities from single-cell transcriptomic data. Each cell was scored against multiple cell death pathway gene sets, and the pathway with the highest score was assigned as the dominant modality. The proportions of cells assigned to each modality was calculated for each cell population.

### 2.2 *In vitro* experiments

NRK-52 E cells were maintained in DMEM (HyClone, Logan, UT, United States of America) containing 10% FBS and antibiotics (100 U/mL penicillin G and 100 μg/mL streptomycin). The cells were incubated at 37 °C in a controlled atmosphere (5% CO_2_, 95% air). Upon reaching approximately 90% confluence, the culture medium was replaced with Hank’s balanced salt solution (HBSS).

### 2.3 Hypoxia-reoxygenation (H/R) model

NRK-52 E cells (90% confluence) were rinsed with PBS and incubated in “glucose-free HBSS (Gibco, 14,175–095) supplemented with 10 μM antimycin A, a mitochondrial complex III inhibitor, for 2 h at 37 °C to induce chemical hypoxia (antimycin A/HBSS model). After 2 h of chemical hypoxia, cells were reoxygenated in complete DMEM. SchB (25 μM) or vehicle (0.1% DMSO) was added at the start of reoxygenation, and the cells were cultured for an additional 24 h. Control cells were not exposed to hypoxic conditions.

### 2.4 Copper ion-induced cell death model

NRK-52 E cells (80%–90% confluence) were divided into four groups: control, CuCl_2_ (1 μM), elesclomol (ES; 100 nM), and CuCl_2_ + ES. The respective treatments were administered for 16 h, and the control cells received no treatment. Cell viability was assessed after culturing in fresh medium. When NRK-52 E cells reached 80% confluence, they were transfected with CTR1-specific or control siRNA using Lipofectamine 2000 (Invitrogen) in Opti-MEM. Three independent siRNA sequences were screened, and the sequence exhibiting the highest knockdown efficiency (>50%), siRNA3 (sense: GGU​UGU​GAU​GUA​CCU​AAG​A; antisense: UCU​UAG​GUA​CAU​CAC​AAC​C), was selected. Successful transfection was validated using western blot analysis. Forty-eight hours after transfection, SchB (25 µM) was added at the onset of reoxygenation and maintained for 24 h, and the cells were subjected to H/R.

### 2.5 Cell viability and cytotoxicity assays

Cell viability was assessed using a CCK-8 kit (Dojindo, Kumamoto, Japan). NRK-52 E cells were seeded in 96-well plates (5 × 10^3^ cells/well). After the designated treatments, CCK-8 solution (10 μL) was added to each well and incubated for 2 h incubation at 37 °C. The absorbance values were recorded at 450 nm using a microplate reader (BioTek, Winooski, VT, United States of America). Cell viability was calculated as a percentage of that of the untreated control group.

Lactate dehydrogenase (LDH) release, a marker of cell membrane damage, was quantified using an LDH Cytotoxicity Assay Kit (Beyotime, Shanghai, China). The culture supernatants were then collected. The LDH assay signal was assessed by recording the absorbance at 490 nm. Total intracellular LDH levels were measured after cell lysis using the provided Lysis Buffer. Cytotoxicity (%) was calculated using the following formula (experimental LDH release − spontaneous LDH release)/(maximum LDH release − spontaneous LDH release) × 100%.

### 2.6 Animals, experimental design

Animal procedures were approved by the Institutional Animal Care and Use Committee (IACUC) of Chongqing Medical University (approval no. IACUC-CQMU-2023–0,156). This study was conducted following the *NIH Guide for the Care and Use of Laboratory Animals* (eighth Edition, 2011).

### 2.7 I/R surgical protocol

Male C57BL/6 J mice (8–12 weeks, 22–25 g, SPF) were purchased from the Experimental Animal Center, Army Medical University (License No. SCXK(Yu)2022-0003). Under general anesthesia induced by intraperitoneal injection of pentobarbital (75 mg/kg), bilateral flank incisions (0.5 cm) were made to expose the renal pedicles. Ischemia was induced using atraumatic microvascular clamps (Roboz, RS-5420) for 28 min at 37 °C ± 0.5 °C. Reperfusion was confirmed by the restoration of kidney color. SchB (50 mg/kg, i. p.) was administered 2 h before ischemia. The sham group was subjected to surgical intervention under the same conditions, while avoiding the clamping step. Body temperature was monitored and maintained at 36.5 °C–37.0 °C using a heating pad (ThermoStar, RWD Life Science). Postoperative pain was managed with subcutaneous buprenorphine (0.1 mg/kg) administered at 12 h intervals.

### 2.8 Renal function assessment

Blood samples were stored at 4 °C for 1 h and centrifuged at 4 °C (3,000 × *g* for 10 min). Serum creatinine (SCr) and blood urea nitrogen (BUN) levels were measured in the supernatants using a Beckman 5,800 Automatic Analyzer (Beckman, Krefeld, Germany). Renal KIM-1 and NGAL mRNA expression was quantified by qPCR as described in the qPCR Methods section.

### 2.9 Quantitative Real-Time PCR (qPCR)

Total RNA was extracted using TRIzol reagent and reverse-transcribed into cDNA using the PrimeScript RT Reagent Kit (Takara, Dalian, China). qPCR was performed on a QuantStudio 5 Real-Time PCR System (Applied Biosystems, Foster City, CA, United States of America) using TB Green Premix Ex Taq II (Takara). A 20 μL qRT-PCR mixture was prepared with 10 μL TB Green Premix, 0.8 μL of each primer (forward and reverse, 10 μM), 2 μL cDNA, and 6.4 μL of nuclease-free water. qRT-PCR was performed at 95 °C for 30 s for initial denaturation and 40 amplification cycles of 95 °C for 5 s and 60 °C for 30 s. Amplification specificity was analyzed using melting curve analysis. Relative gene expression was calculated using the 2^−ΔΔCT^ method. β-actin was used as the internal reference gene for both rat (NRK-52 E) and mouse (kidney tissue) samples. All the reactions were performed in triplicates. The mRNA levels of kidney injury molecule-1 (KIM-1) and neutrophil gelatinase-associated lipocalin (NGAL) were determined using qPCR.

KIM-1(F) 5′-AAG​GTT​GGT​GGA​GAG​GCA​GGT​C-3'.

KIM-1(R) 5′-TCC​GAT​AGG​TGA​CGT​GGG​TTC​C-3'.

NGAL(F) 5′-GCA​GCA​GAA​CTT​CCA​GGA​CAA​CC-3'.

NGAL(R) 5′-CAG​GAC​GGA​GGT​GAC​ATT​GTA​GC-3'.

### 2.10 Oxidative stress biomarker detection

Intracellular malondialdehyde (MDA) levels were measured using a thiobarbituric acid reactive substance (TBARS) assay kit (Beyotime, Shanghai, China). Briefly, cells or homogenized kidney tissues were lysed and incubated with thiobarbituric acid (95 °C, 60 min). Following centrifugation, the absorbance of the supernatant was measured at 532 nm. MDA levels were quantified using a standard curve and normalized to the corresponding total protein concentrations.

The intracellular GSH content was quantified using a GSH assay kit (Nanjing Jiancheng Bioengineering Institute, Nanjing, China) based on the chromogenic reaction between GSH and 5,5′-dithiobis (2-nitrobenzoic acid) (DTNB). The absorbance was measured at 412 nm. GSH levels were normalized to the total protein concentration. Superoxide dismutase 2 (SOD2) activity was assessed using Cu/Zn-SOD and Mn-SOD assay kits (Beyotime, Shanghai, China). Enzyme activity was expressed as U/mg of protein.

### 2.11 Study subjects and pathological data

Clinical and pathological data from patients with AKI diagnosed between January 1 and 30 December 2021, were retrospectively reviewed using electronic medical records and Renal Pathology Archives. Histological evaluation of AKI was based on light microscopic detection of necrotic and detached/sloughed RTECs, and electron microscopy was used to validate ultrastructural alterations. The control samples consisted of histologically normal renal tissues adjacent to the renal cell carcinoma, with minimal pathological changes. Two nephrologists independently re-evaluated the specimens. This study was reviewed and approved by the Ethics Committee of the Third Affiliated Hospital of Chongqing Medical University (Approval No. 2022–104).

### 2.12 RNA sequencing and bioinformatics analysis

Total RNA was extracted from NRK-52 E rat cells using TRIzol reagent (Invitrogen, Carlsbad, CA, United States of America). RNA integrity was assessed using an Agilent Bioanalyzer 2,100 (Agilent Technologies, Santa Clara, CA, United States of America). High-quality RNA samples (RIN ≥7.0) were used for transcriptome analysis. Strand-specific mRNA libraries were prepared using the NEBNext Ultra II RNA Library Prep Kit (New England Biolabs, Ipswich, MA, United States of America). Libraries were sequenced on an Illumina NovaSeq 6,000 instrument to produce 150-bp paired-end reads.

Raw reads were filtered using fastp (v0.23.2) to remove adapter sequences and low-quality bases. Clean reads were aligned to the *Rattus norvegicus* reference genome (mRatBN7.2) using HISAT2 (v2.2.1). Gene-level read counts were obtained using featureCounts (v2.0.1; -p​ for paired-end libraries and -s 2​ for the strand-specific protocol). Differential expression analysis between groups was performed using DESeq2 (v1.38.0) in R (v4.2.1), which was applied to the raw count matrix and incorporates median-of-ratios normalization. Genes with |log_2_ fold change| ≥ 1.0 and adjusted P-value (Benjamini–Hochberg false discovery rate, FDR) < 0.05 were defined as differentially expressed genes (DEGs). GO and KEGG pathway enrichment analyses were performed using clusterProfiler (v4.6.0), and visualization was performed using the ggplot2 and ComplexHeatmap packages.

### 2.13 Western blot analysis

Total protein was extracted from NRK-52 E cells and mouse kidney tissues, and protein concentrations were determined using a BCA protein assay kit. SDS sample buffer was added, and the samples were mixed thoroughly and heated at 100 °C for 10 min to denature the proteins. Equal amounts of protein (30–80 μg) were separated on 12% SDS-polyacrylamide gels and transferred onto PVDF membranes at 260 mA for 90min. Membranes were blocked with 5% bovine serum albumin in Tris-buffered saline containing 0.1% Tween-20 (TBST) for 1 h at room temperature, followed by three washes with TBST for 5 min each. For targets of widely separated molecular weights, membranes were cut horizontally into strips according to molecular weight range and each strip was probed with a single primary antibody; strips were reassembled for presentation, and white space indicates where strips from the same membrane have been joined. Because DLAT and HSP70 migrate at closely similar molecular weights (∼70 kDa) and cannot be resolved as distinct bands on a 12% gel, the corresponding strip was probed sequentially: following detection of DLAT, the strip was stripped and reprobed for HSP70. Membranes were incubated overnight at 4 °C on a shaker with the following primary antibodies: CTR1 (1:1,000, Cat#. 13086S, Cell Signaling Technology, Danvers, MA, United States of America), FDX1 (1:1,000, Cat#. NBP1-89227 (Novus, Colorado Springs, CO, United States of America), and β-actin (1:2,000, Cat#. T40104 S (AbMart, Shanghai, China) was added and incubated overnight on a shaker at 4 °C. Horseradish peroxidase-conjugated anti-mouse IgG (1:3,000, Catalog #. 7076S, Cell Signaling Technology) and horseradish peroxidase-conjugated goat anti-rabbit IgG (1:10,000, Cat# 7074, RRID: AB_2099233, Cell Signaling Technology) secondary antibodies were added to the membranes. ImageJ software was used to quantify band intensities, which were normalized to the loading control β-actin.

### 2.14 Copper concentrations in the cells

HNO3 (0.5 mL) and H_2_O_2_ (0.5 mL) were added to the cell pellets. The samples were heated and digested at 120 °C for 4 h, diluted with ultrapure water to a final volume of 5 mL, and homogenized after the solution reached room temperature. Quantitative analysis of copper content was performed by Beijing Zhongke Baise Testing Technology Co., Ltd. Using a NexION 300D ICP-MS instrument (PerkinElmer, Waltham, MA, United States of America).

### 2.15 Immunohistochemistry and immunofluorescence staining

After 24 h of fixation in 10% neutral buffered formalin, renal tissues were processed, embedded, and cut into 4-μm sections. The sections were then dewaxed and rehydrated. Following the addition of EDTA buffer (pH 8.0), antigen retrieval was performed under high temperature and pressure for 120 s. Goat serum was added and incubated for 30 min, followed by incubation with FDX1 antibody (1:1,000) for at least 12 h. Subsequently, an HRP-labeled goat anti-rabbit/mouse secondary antibody (Cat#. PV-8000 (Origene, Beijing, China) was added and incubated for 40 min. For immunofluorescence staining, an FITC-labeled goat anti-rabbit secondary antibody (1:50, Cat#. SA00003-2 (Proteintech Group, Wuhan, China) was used. After washing and mounting, the sections were observed using fluorescence microscopy. The sections were examined using an DS-i2 light microscope (Nikon, Tokyo, Japan).

Two nephrologists independently reviewed the samples under blinded conditions to minimize assessment bias in the study. At least 10 non-overlapping visual fields per sample were examined at ×200 magnifications. The percentage of CTR1 and FDX1-positive renal tubules was calculated relative to the total number of renal tubules examined per section.

### 2.16 Histologic examinations

Tissue sections (4 µm) were stained with periodic acid–Schiff and Masson’s trichrome and analyzed under a Nikon microscope (Nikon, Tokyo, Japan). The extent of renal tubular injury was quantified from no fewer than 10 randomly selected fields per sample, with scoring performed independently by two nephrologists who were unaware of sample allocation. Tubular injury was graded according to the percentage of tubules in the outer medulla exhibiting epithelial necrosis and/or necrotic debris: score 0, <10%; score 1, 10%–25%; score 2, 26%–50%; score 3, 51%–75%; and score 4, >75%.

### 2.17 Molecular docking and validation

The three-dimensional structure of SchB (PubChem CID: 108130) was retrieved from the PubChem database and energy-minimized using ChemBio3D Ultra. The gene and amino acid sequence information of human CTR1 (encoded by SLC31A1) were obtained from the UniProt database and RCSB PDB database (Wen et al., 2025). The receptor structure was preprocessed using PyMOL (v2.6.0), which comprised the removal of the co-crystallized ligand, crystallographic water molecules, and redundant protein chains, followed by the addition of hydrogen atoms. Both the receptor and the ligand were then converted into the PDBQT format using AutoDock Tools (v1.5.7), with polar hydrogen atoms added and Kollman partial atomic charges assigned. A grid box with dimensions of 60 × 60 × 60 Å and a spacing of 0.375 Å was centered on the putative copper-binding site of CTR1. Molecular docking of SchB to CTR1 was performed using AutoDock Vina (v1.1.2) with an exhaustiveness parameter of 20 and 20 independent docking runs conducted to ensure the reliability of the docking results (Ma et al., 2024). The conformation with the lowest binding energy was selected and visualized using PyMOL (v2.6.0).

### 2.18 Statistical analysis

Results are expressed as mean ± SD from a minimum of three independent replicates per experiment. Data normality was assessed using the Shapiro–Wilk test. For two-group comparisons, an unpaired t-test (for normally distributed data) or the Mann–Whitney U test (for non-normally distributed data) was applied. For comparisons involving more than two groups, one-way ANOVA with Tukey’s correction or Games–Howell *post hoc* analysis was used, depending on variance homogeneity (tested by Levene’s test). Pearson correlation analysis was used for normally distributed biomarkers, whereas Spearman correlation analysis was applied for non-normally distributed variables. All statistical analyses were performed using GraphPad Prism 8, with statistical significance defined as P < 0.05.

## 3 Results

### 3.1 The involvement of cuproptosis in AKI occurrence and development

#### 3.1.1 Single-cell transcriptomic profiling identifies cuproptosis as a predominant regulated cell death modality in AKI

Single-cell RNA sequencing data from the GSE180420 dataset (*n* = 110,000 cells) were analyzed. Cell type annotation identified the major renal compartments, including the proximal convoluted tubule (PCT), proximal straight tubule (PST), loop of Henle (LOH), and distal convoluted tubule (DCT) (Figure 1A). Pathway gene set scoring of tubular epithelial cells identified cuproptosis as the most prominent regulated cell death modality, accounting for approximately 33% of the cells assigned to the regulated cell death pathway (Figure 1B). Notably, cuproptotic cell death was specifically enriched in the renal tubular epithelial cell population. Cuproptosis was specifically enriched in RTECs (PCT/PST) compared to other nephron segments (Figure 1C). Compared with the sham-operated control group, the proportion of cuproptosis increased significantly after ischemic injury (Figure 1D). GO analysis revealed significant enrichment of cuproptosis-related biological processes after I/R injury, particularly those involving oxidative stress responses, cell-cell signaling by Wnt, regulation of Wnt signaling, stress-activated MAPK cascade, JAK-STAT (Figure 1E). KEGG pathway analysis highlighted metal ion homeostasis and MAPK signaling as the most significantly altered pathways (Figure 1F), implicating copper-redox dysregulation in AKI pathogenesis.

**FIGURE 1:**
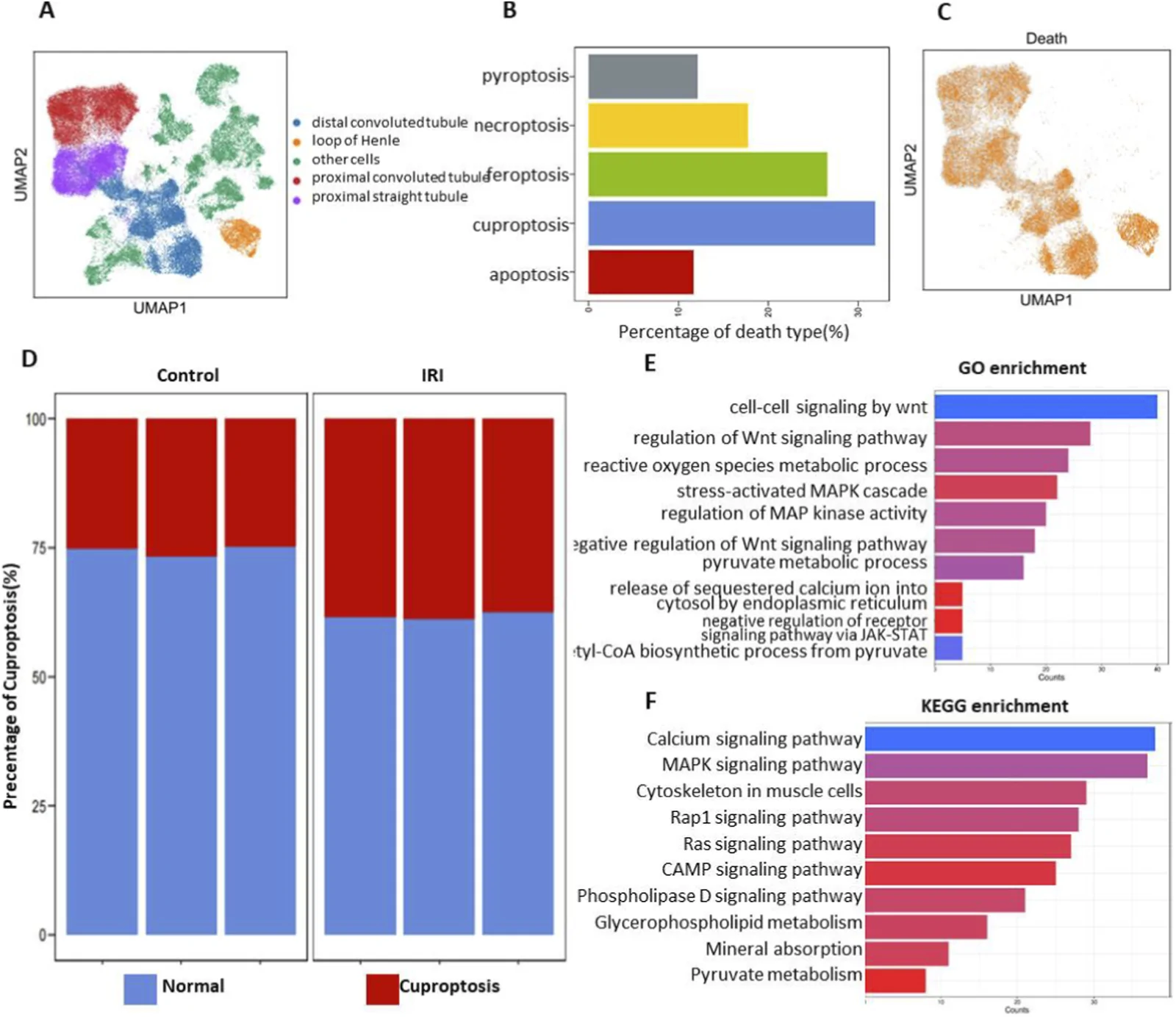
Cuproptosis is a critical form of programmed cell death in AKI. **(A–C)** Quantitative reverse-deconvolution analysis of cell death patterns revealed that cuproptosis was a predominant mechanism. **(D)** Compared with the control group, the proportion of cuproptosis increased significantly after IR. **(E,F)** Gene Ontology (GO) and KEGG pathway analyses confirmed the specific enrichment of cuproptosis-related pathways; metal ion regulation and reactive oxygen species (ROS) metabolism were the most significantly altered metabolic cascades. Data are presented as proportions or enrichment scores as indicated. Comparisons between two groups were performed using an unpaired Student’s t-test or the Mann–Whitney U test, as appropriate. **P < 0.01 vs. Sham group. Statistical significance was defined as P < 0.05.

#### 3.1.2 Upregulation of CTR1 and FDX1 in human AKI kidney tissues

CTR1 (encoded by *SLC31A1*) is the principal transporter of copper into cells, whereas FDX1 is a key regulator of cuproptosis. To further assess the clinical relevance of cuproptosis in AKI, we included 22 patients diagnosed with AKI according to the KDIGO criteria and six age-matched controls whose normal tissue was obtained adjacent to tumors duting nephrectomy. Samples were collected at the Third Affiliated Hospital of Chongqing Medical University between January and December 2021 (Table 1). Although the cohort included only one case of isolated ischemic AKI, most cases were associated with nephrotoxic exposures, toxins, or infection. Immunohistochemical staining showed increased CTR1 and FDX1 expression in tubular epithelial cells across these etiologies, suggesting that cuproptosis may be involved in tubular injury pathway beyond ischemic AKI Baseline characteristics demonstrated elevated SCr (AKI group vs. control group: 481.7 ± 255.4 vs. 69.2 ± 14.6 μmol/L, P < 0.01), elevated blood urea nitrogen (BUN; 18.37 ± 9.63 vs. 5.27 ± 1.03 mmol/L, P = 0.0029), and reduced estimated glomerular filtration rate (eGFR; 16.6 ± 15.6 vs. 106.7 ± 24.8 mL/min/1.73 m^2^, **P < 0.01) in the AKI cohort (Figures 2A–C). Urinary N-acetyl-β-D-glucosaminidase (NAG), urinary retinol-binding protein (URBP) and urinary transferrin (UTRF) levels were increased in the AKI group (Figures 2D–F). Immunohistochemistry localized CTR1 and FDX1 predominantly to the plasma membrane and perinuclear regions of tubular epithelial cells (Figure 3A). Quantitative morphometry revealed dramatic upregulation in AKI tissues: CTR1 (24.96% ± 7.77% vs. 2.05% ± 0.86%, **P < 0.01) and FDX1 (33.44% ± 9.96% vs. 2.53% ± 0.96%, **P < 0.01) (Figures 3B,C). Significant positive associations were observed between CTR1 expression and injury biomarkers: SCr (r = 0.899, **P < 0.01), BUN (r = 0.723, **P < 0.01), and urinary N-acetyl-β-D-glucosaminidase (NAG; r = 0.701, **P < 0.01), with an inverse correlation with eGFR (r = −0.860, **P < 0.01) (Figures 3D–G). FDX1 exhibited parallel correlations, validating the utility of cuproptosis markers in reflecting the severity of tubular injury (Figures 3H–K).

**TABLE 1:** Clinical, renal pathological, and CTR1/FDX1 expression data for 22 AKI patients and six age-matched controls.

Number	Sex	Age	Scre	Bun	eGFR	NAG	URBP	UTRF	Cause of AKI	CTR1	FDX1
-	-	(years)	(μmol/L)	(mmol/L)	(ml/min/1.73m^2^)	(U/L)	(mg/L)	(mg/L)	-	(%)	(%)
AKI1	F	47	300	13.3	15.4	22.2	1.47	1.96	Antibiotic	20.2	29.4
AKI2	M	30	112	5.18	70	23.1	2.95	2.69	NASID	16.8	18.5
AKI3	F	45	646	7.92	6.16	44.1	5.66	11.59	Antibiotic and NSAID	28.5	35.2
AKI4	M	79	271	9.19	21	82.8	7.93	9.66	Infection	17.8	23.9
AKI5	M	34	692	38.67	8.13	18.9	6.49	12.96	Biotoxin	24.6	39.7
AKI6	M	64	1,195	34.83	4	86.4	​	​	Unknown	38.5	55.8
AKI7	M	63	691	24.75	6.64	30.4	10.3	4.26	Antiviral and antibiotic	37.7	48.1
AKI8	F	52	461	17.16	9.2	​	​	​	Infection	28.9	36.6
AKI9	F	56	806	37.72	4.36	53.1	10.32	14.88	Unknown	39.4	40.3
AKI10	M	71	168	6.22	37.3	17.8	0.51	1.07	NSAID	16.1	23.8
AKI11	F	63	711	14.46	5.4	52.2	4.24	23.17	Biotoxin	30.6	41.9
AKI12	M	40	431	17.92	13.81	18.1	1.75	3.87	NSAID	21.3	22.4
AKI13	F	32	267	23.83	19.64	12.9	3.23	6.8	Biotoxin	16.4	25.5
AKI14	M	54	156	6.12	43	13.4	0.71	0.5	Antiviral and antibiotic	14.7	19.6
AKI15	M	29	688	18.7	8.48	46.1	94.1	23.9	Chemical toxins	34.2	42.7
AKI16	M	56	485	24.4	10.7	18.4	4.7	7.8	Biotoxin	19.6	39.8
AKI17	F	88	553	15.5	5.5	33.9	18.64	6.4	Antibiotic	24.8	40.4
AKI18	M	66	346	12.9	15	19.2	1.9	10.34	Antibiotic	18.9	23.5
AKI19	M	58	449	14.5	11.58	26.5	2.31	3.45	Infection	25.4	29.3
AKI20	M	30	522	20.6	11.72	37.3	4.37	14.97	Biotoxin	32.5	40.4
AKI21	M	31	290	17.8	23.75	19.9	3.94	6.29	Unknown	19.7	25.7
AKI22	M	74	358	22.4	13.61	20.4	2.96	3.44	Ischemia	22.6	33.1
C1	M	28	79	4.6	116.84	4.1	0.2	1.14	-	1.8	2.8
C2	F	47	46	5.5	148.18	6.6	0.5	2.05	-	2.7	3.4
C3	F	55	72	3.9	81.5	5.9	0.9	0.96	-	3.2	1.2
C4	M	64	83	4.8	84.88	7.4	1.1	1.48	-	1.5	2.3
C5	F	49	57	6.1	112.75	8.5	0.8	2	-	2.3	1.8
C6	M	58	78	6.7	96.11	9.0	0.1	1.08	-	0.8	3.7

**FIGURE 2:**
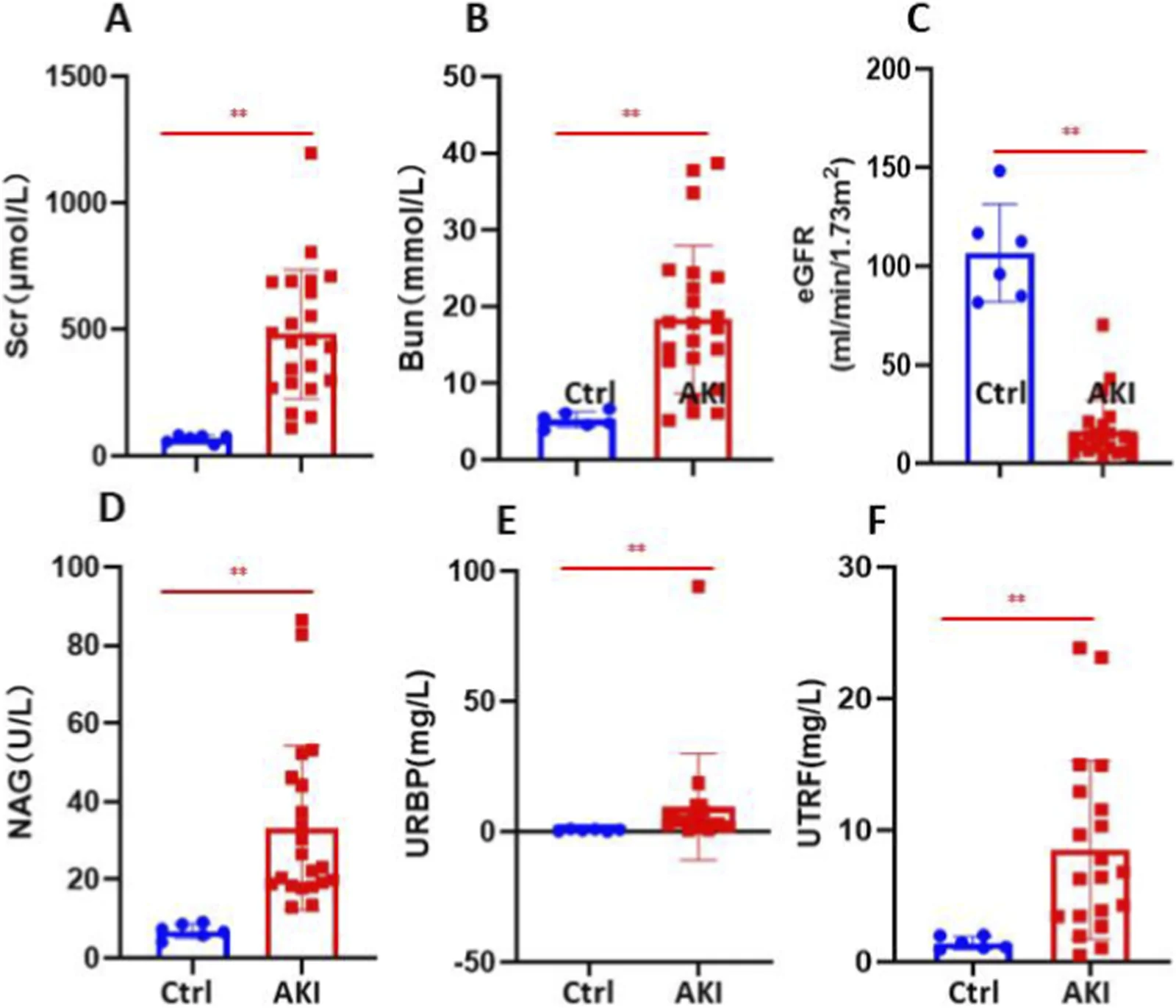
Comparison of clinical and pathological characteristics between AKI patients and controls. **(A)** Serum creatinine (Scr) levels in the Ctrl and AKI models. **(B)** Blood urea nitrogen (Bun) levels in the Ctrl and AKI models. **(C)** Estimated glomerular filtration rate (eGFR) in the Ctrl and AKI models. **(D)** N-acetyl-β-D-glucosaminidase (NAG) levels in the Ctrl and AKI models. **(E)** Urinary retinol-binding protein (URBP) levels in the Ctrl and AKI models. **(F)** Urinary transferrin (UTRF) levels in the Ctrl and AKI models. **Data are presented as mean ± SD. n = 28 (Ctrl = 6 and AKI = 22 patients). Comparisons between two groups were performed using an unpaired Student’s t-test. **P < 0.01 vs. Control group. Statistical significance was defined as P < 0.05.

**FIGURE 3:**
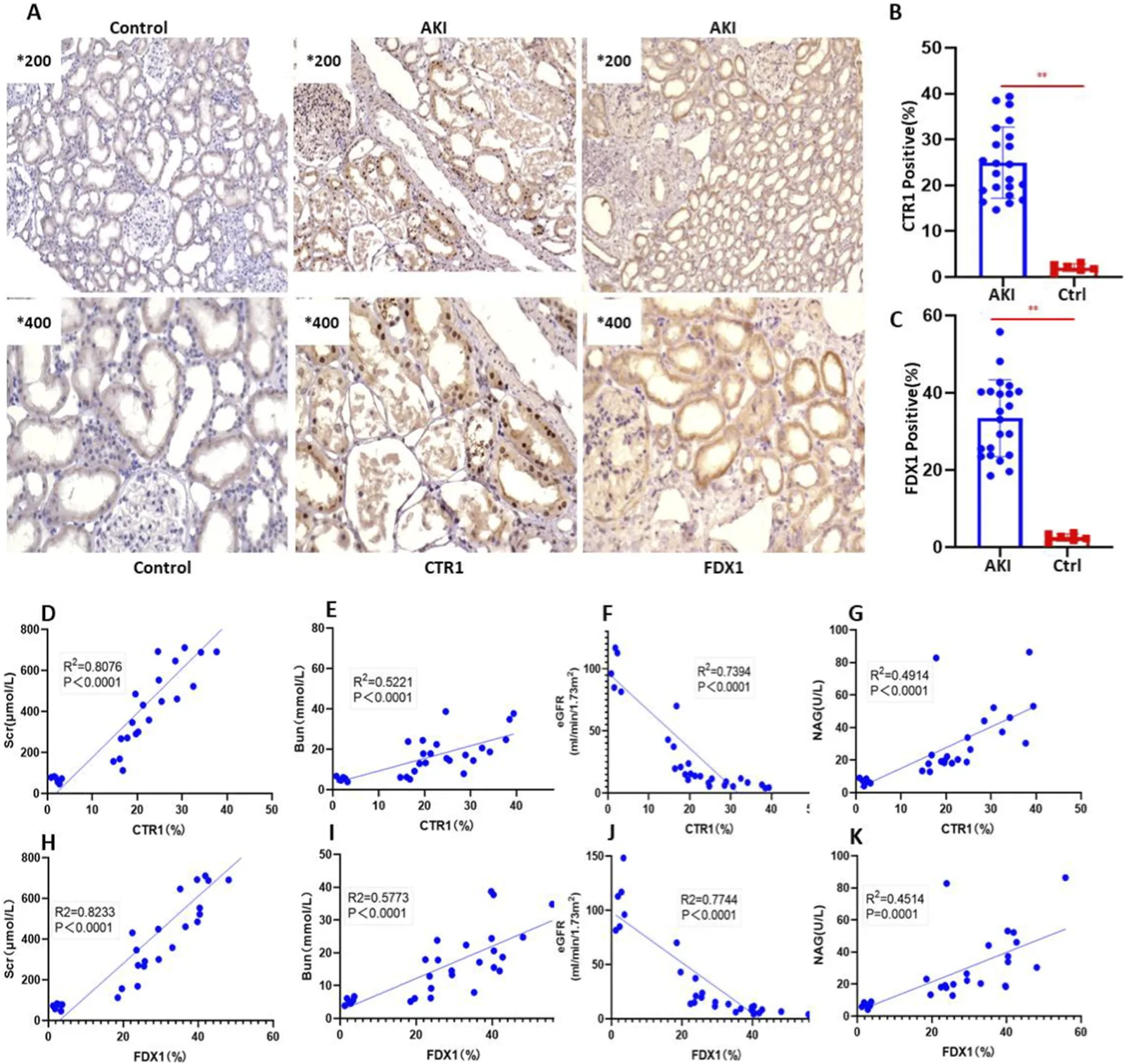
Cuproptosis in the kidney tissue of patients with AKI. **(A)** Representative immunohistochemical staining of CTR1 and FDX1 in Control (n = 6) and AKI groups (n = 22) (magnifications:×200 and ×400). FDX1 and CTR1 were mainly expressed in the plasma membrane and perinuclear regions of tubular epithelial cells.​ **(B,C)** Quantitative analysis of CTR1 and FDX1 positive areas (%). Data are expressed as mean ± SD. Comparisons between two groups were performed using an unpaired Student’s t-test. **P < 0.01 vs. Control group. **(D–K)** Correlation analyses of CTR1 and FDX1 expression with clinical indicators. CTR1 and FDX1 expression was positively correlated with serum creatinine (Scr), blood urea nitrogen (Bun), and NAG levels, and negatively correlated with estimated glomerular filtration rate (eGFR) levels. Correlation analyses were performed using Pearson’s correlation or Spearman’s correlation. All statistical analyses were performed using GraphPad Prism 8, with statistical significance defined as *P* < 0.05.

#### 3.1.3 Copper accumulation and cuproptosis activation in the renal tissue of AKI mice

To establish temporal dynamics, C57BL/6 J mice underwent bilateral renal ischemia (28 min) followed by 48 h of reperfusion (*n* = 6 mice). Histological examination revealed characteristic AKI pathology, including tubular epithelial vacuolization, brush border loss, cast formation, and interstitial inflammation (Figure 4A; PAS staining). Renal function deteriorated markedly, as indicated by elevated SCr (206.5 ± 31.8 vs. 31.3 ± 6.9 μmol/L, **P < 0.01) and BUN levels (16.0 ± 2.5 vs. 4.8 ± 1.3 mmol/L, **P < 0.01) (Figures 4B,C). Tubular injury scores confirmed severe damage (2.8 ± 0.6 vs. 0.3 ± 0.5, **P < 0.01) (Figure 4D). Tissue copper quantification by inductively coupled plasma mass spectrometry (ICP-MS) demonstrated a five-fold increase in kidney copper levels (4.8 ± 1.2 vs. 0.9 ± 0.23 μg/g, **P < 0.01) (Figure 4E), coinciding with the marked upregulation of CTR1 and FDX1 expression (Figures 4F–H). Western blot analysis confirmed increased expression of CTR1, FDX1, DLAT, and HSP70 compared with sham controls (**P < 0.01) (Figures 4I–M), thereby establishing the I/R model for subsequent mechanistic studies.

**FIGURE 4:**
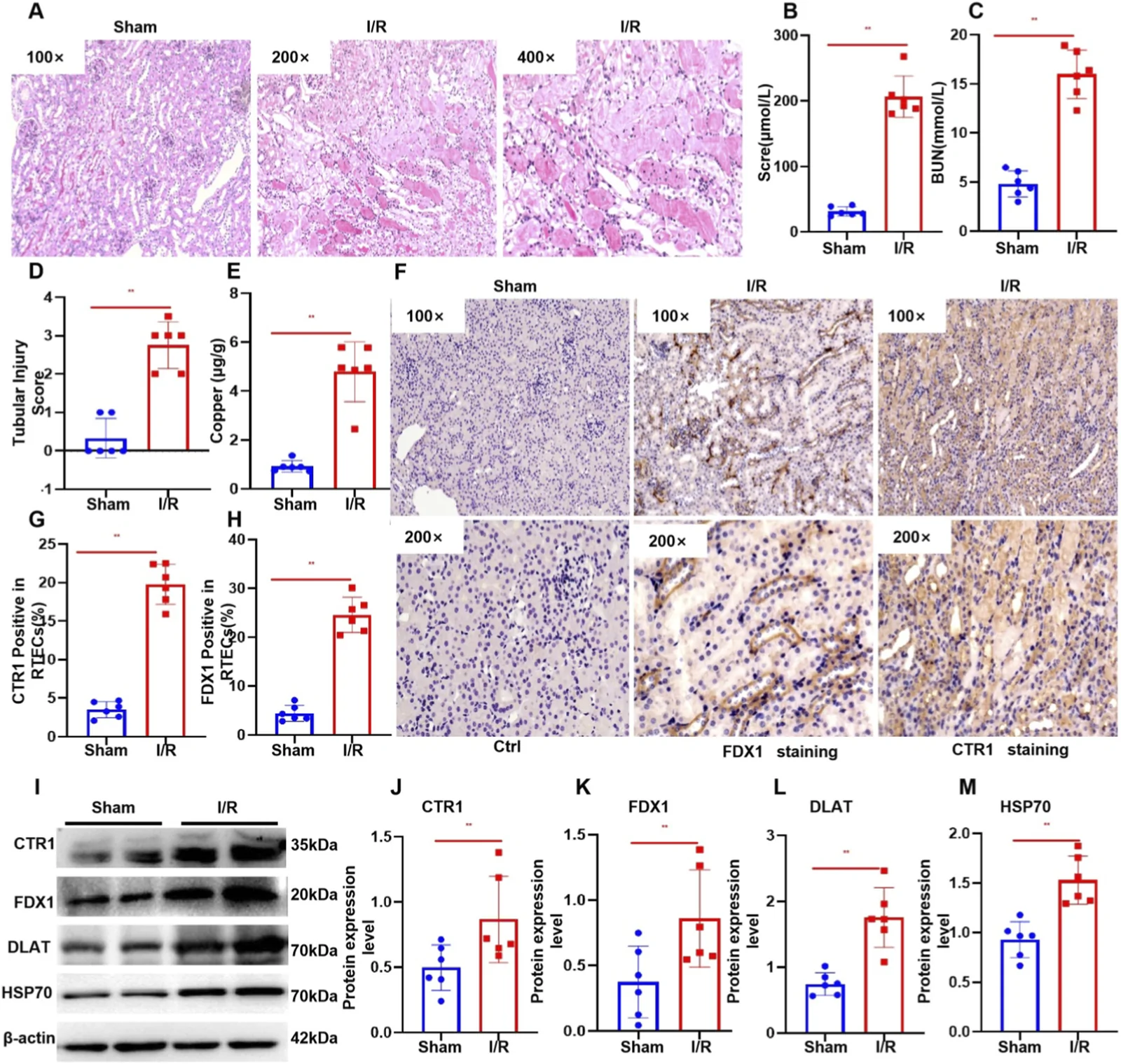
Cuproptosis in the renal tissue of AKI mice. **(A–D)** AKI mice showed significant damage to RTECs, with increased serum creatinine and urea nitrogen levels. **(E)** Kidney copper concentration was significantly increased in I/R mice. **(F–H)** FDX1 and CTR1 were specifically expressed in the plasma membrane and perinuclear regions of mouse RTECs. **(I–M)** Western blot analysis confirmed the significant upregulation of CTR1, FDX1, DLAT, and HSP70 in the kidneys of AKI mice. Data are expressed as mean ± SD (n = 6 per group). Comparisons between two groups were performed using an unpaired Student’s t-test. **P < 0.01 vs. Sham group. Statistical significance was defined as P < 0.05.

#### 3.1.4 Establishment of an *in vitro* copper overload model

To directly assess copper-dependent cytotoxicity, NRK-52 E cells were treated with CuCl_2_ (1 μM), elesclomol (ES; a copper ionophore, 100 nM), or a combination of both, for 16 h. While individual treatments showed minimal toxicity, the combination of CuCl_2_ and ES induced marked cell death: LDH release increased 4.3-fold (*P* < 0.01), cell viability decreased to 38.2% ± 4.1% (P < 0.01), and intracellular copper levels increased 2.1-fold (*P* < 0.01) (Figures 5A,B). Injury markers NGAL and KIM-1 were significantly elevated (**P < 0.05) (Figures 5C,D), accompanied by upregulation of CTR1 and FDX1 (*P < 0.05) (Figures 5E–G), confirming copper-dependent RTEC death via cuproptosis.

**FIGURE 5:**
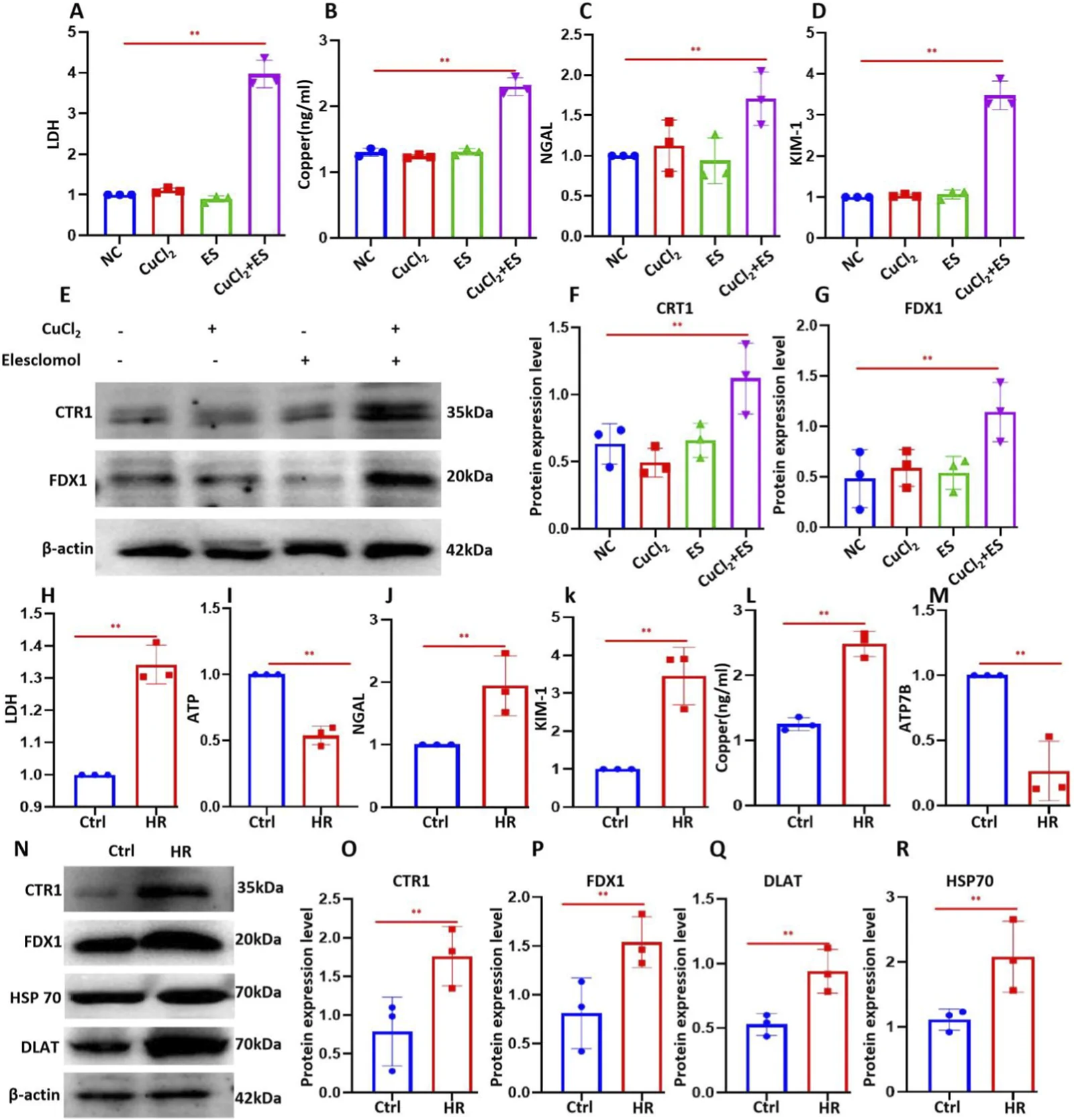
Effect of copper and elesclomol on RTECs. **(A,B)** LDH and intracellular copper levels were significantly increased, and cell viability was significantly decreased in the CuCl_2_ + ES group. **(C,D)** NGAL and KIM-1 levels were markedly increased in the CuCl_2_ + ES group. **(E–G)** The expression of CTR1 and FDX1 was markedly increased in the CuCl_2_ + ES group. **(H)** LDH release in the H/R-induced RTEC model. **(I)** ATP levels in the H/R RTEC model. **(J,K)** NGAL and KIM-1 expression in the H/R RTEC model. **(L)** Copper ion concentration in the H/R RTEC model. **(M)** ATP7B expression in the H/R RTEC model. **(N–R)** CTR1, FDX1, HSP70, and DLAT expression in the H/R RTEC models. Data are expressed as mean ± SD from a minimum of three independent replicates per experiment (n = 3 per group). Comparisons between two groups were performed using an unpaired Student’s t-test or the Mann–Whitney U test, as appropriate. *P < 0.05, **P < 0.01 vs. indicated groups. Statistical significance was defined as P < 0.05.

#### 3.1.5 Cuproptosis-related factor expression in H/R-challenged RTECs

The H/R challenge (2 h hypoxia/2 h reoxygenation) recapitulated the findings observed in the I/R model, including elevated LDH release (**P < 0.01), ATP depletion (48.6% reduction, *P < 0.05), upregulation of NGAL and KIM-1 (*P < 0.05), and copper accumulation (2.2-fold increase, **P < 0.01) (Figures 5H–L). The expression of ATP7B, a copper exporter, decreased by 68% (*P < 0.05) (Figure 5M), suggesting impaired copper clearance in the kidney. Cuproptosis-related proteins and stress-responsive markers, including CTR1, FDX1, DLAT, and HSP70, were significantly induced (*P < 0.05) (Figures 5N–R), establishing H/R as a relevant *in vitro* model of cuproptosis.

### 3.2 Schisandrin B attenuates cuproptosis and confers renoprotection in AKI

SchB, a bioactive lignan derived from *S. chinensis*, possesses anti-inflammatory and antioxidant properties, promotes mitochondrial repair, and attenuates renal fibrosis by inhibiting epithelial–mesenchymal transition (EMT). Based on our finding that cuproptosis is a key mechanism underlying I/R-induced AKI, we hypothesized that SchB attenuates cuproptosis in AKI. Therefore, we investigated whether SchB reduces renal injury by inhibiting cuproptosis.

#### 3.2.1 SchB protects against H/R-induced tubular epithelial cell injury *in vitro*

SchB (25 μM) was added at the beginning of the 2 h reoxygenation phase and was maintained for an additional 24 h. Under these conditions, SchB significantly ameliorated H/R-induced injury: NGAL and KIM-1 mRNA levels were reduced by 71% and 68%, respectively (*P* < 0.01), and LDH release decreased by 54% (*P* < 0.01) (Figures 6A–C). Intracellular copper levels were normalized (**P < 0.01), accompanied by downregulation of CTR1, FDX1, DLAT, and HSP70 (**P < 0.01) (Figures 6D–I), indicating potent inhibition of cuproptosis.

**FIGURE 6:**
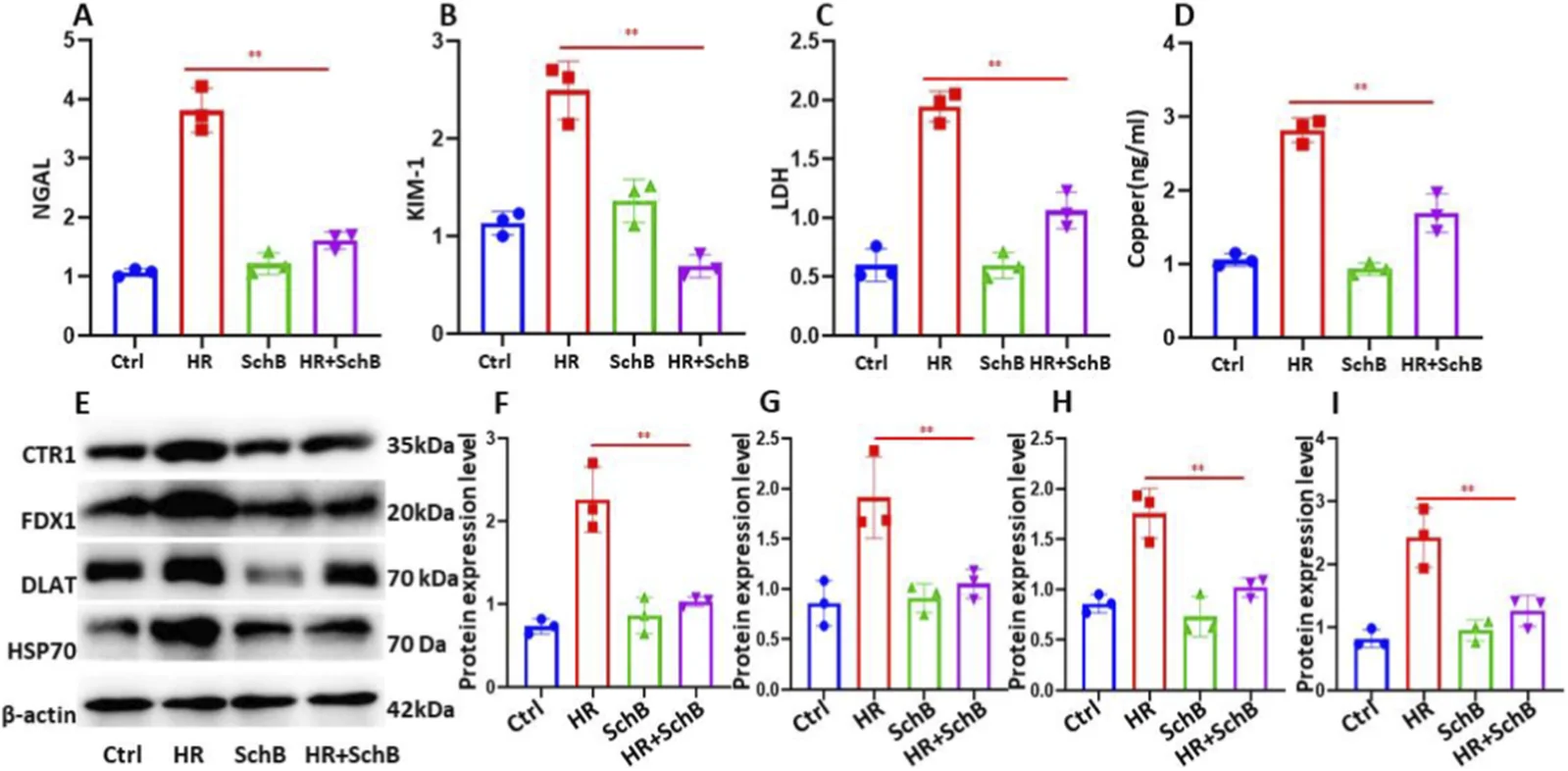
SchB significantly inhibits HR-induced tubular epithelial cell death. **(A–C)** SchB administration suppressed H/R-induced upregulation of NGAL, KIM-1, and LDH. **(D–I)** SchB administration suppressed H/R-induced upregulation of Cu concentrations and cuproptosis-associated proteins, including CTR1, FDX1, HSP70, and DLAT. Data are expressed as mean ± SD from a minimum of three independent replicates per experiment (n = 3 per group). Comparisons between two groups were performed using an unpaired Student’s t-test. **P < 0.01 vs. indicated groups. Statistical significance was defined as P < 0.05.

#### 3.2.2 SchB treatment significantly improves renal function in I/R-induced AKI mice

To validate the renoprotective effects of SchB *in vivo*, we used an I/R-induced AKI mouse model. SchB administration (50 mg/kg, i. p., 2 h pre-ischemia) produced no detectable alterations in renal function or histology in sham-operated mice (Figures 7A–C). SchB significantly improved renal function, as evidenced by marked reductions in BUN and SCr levels compared to the I/R vehicle group (**P < 0.01) (Figures 7B,C). Histological protection included reduced tubular necrosis, diminished cast formation, and preservation of the brush border (injury score: 1.25 ± 0.46 vs. 2.75 ± 0.61, **P < 0.01) (Figure 7D). At the molecular level, SchB suppressed NGAL and KIM-1 expression (**P < 0.01) (Figures 7E,F), reduced tissue copper content (**P < 0.01) (Figure 7G), and downregulated cuproptosis-related proteins (**P < 0.01) (Figures 7H–L). SchB effectively ameliorated I/R-induced renal dysfunction and tubular injury by suppressing cuproptosis.

**FIGURE 7:**
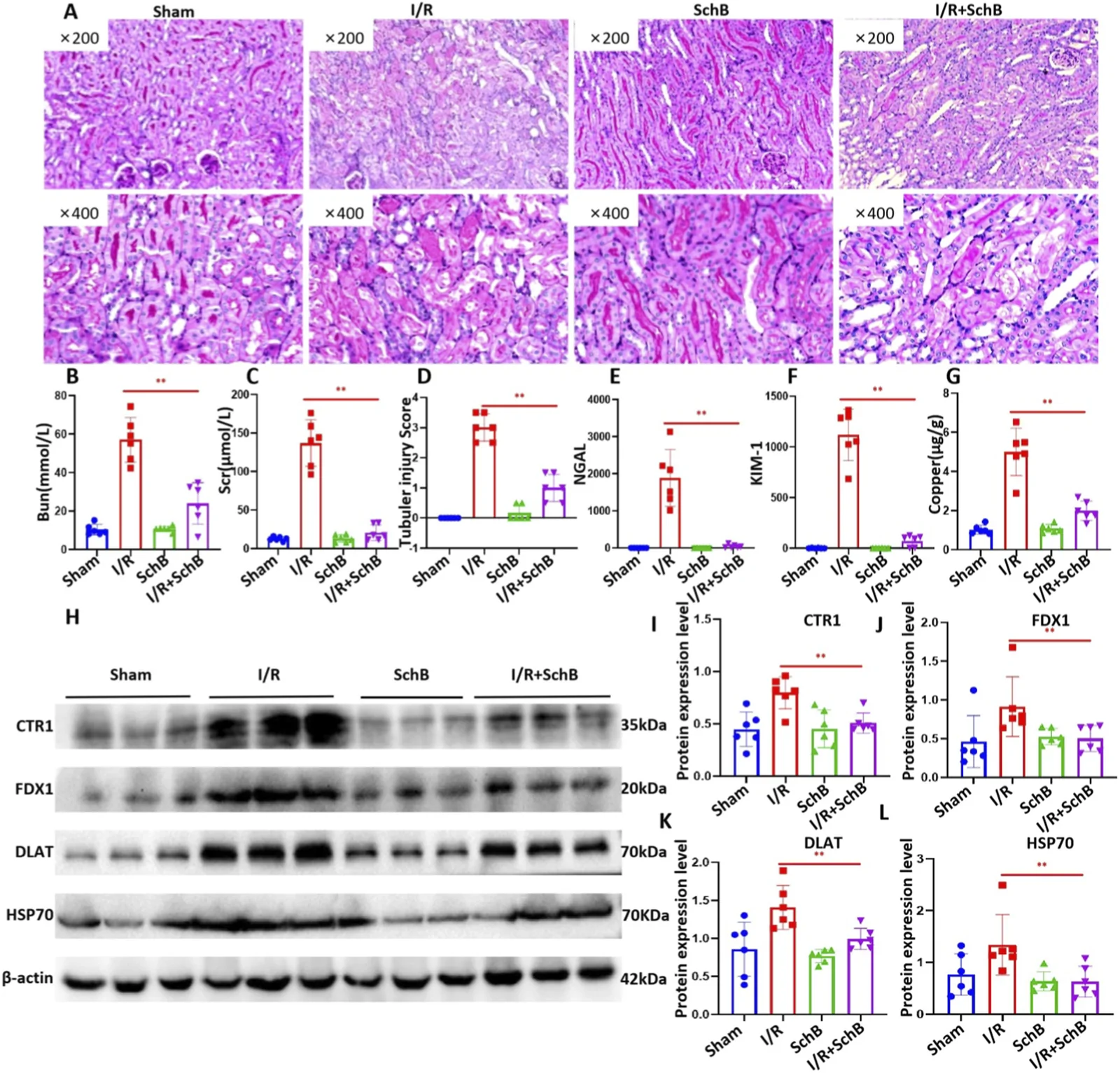
SchB treatment significantly improves renal function in I/R-induced AKI mice. **(A–C)** SchB demonstrated an excellent safety profile, and SchB treatment significantly reduced BUN and creatinine levels in I/R-induced AKI in mice. **(D–F)** SchB provided significant protection to the renal tissue structure and suppressed the injury-induced upregulation of the tubular markers NGAL and KIM-1. **(G–L)** SchB administration suppressed copper accumulation and cuproptosis-associated proteins and stress-responsive marker, including CTR1, FDX1, DLAT, and HSP70, in I/R-induced AKI mice. Data are expressed as mean ± SD from a minimum of three independent replicates per experiment (n = 3 per group). Comparisons among multiple groups were performed using one-way ANOVA with Tukey’s *post hoc* test. **P < 0.01 vs. indicated groups. Statistical significance was defined as P < 0.05.

#### 3.2.3 SchB inhibits cuproptosis by reducing oxidative stress and regulating copper metabolism

RNA sequencing of H/R-challenged RTECs ± SchB identified 1,581 DEGs, including 513 upregulated and 1,068 downregulated genes following SchB treatment (Figures 8A,B). KEGG enrichment analysis highlighted glutathione metabolism, p53 signaling, cellular senescence, and the PI3K-Akt pathway (Figure 8C), which is consistent with antioxidant activity and promotion of cell survival. SchB reduced MDA levels, a marker of lipid peroxidation, by 61% *in vitro* and 48% *in vivo* (**P < 0.01), while increasing GSH levels by 2.3-fold and 1.9-fold, respectively (**P < 0.01) (Figures 8D–G), confirming its potent antioxidant activity.

**FIGURE 8:**
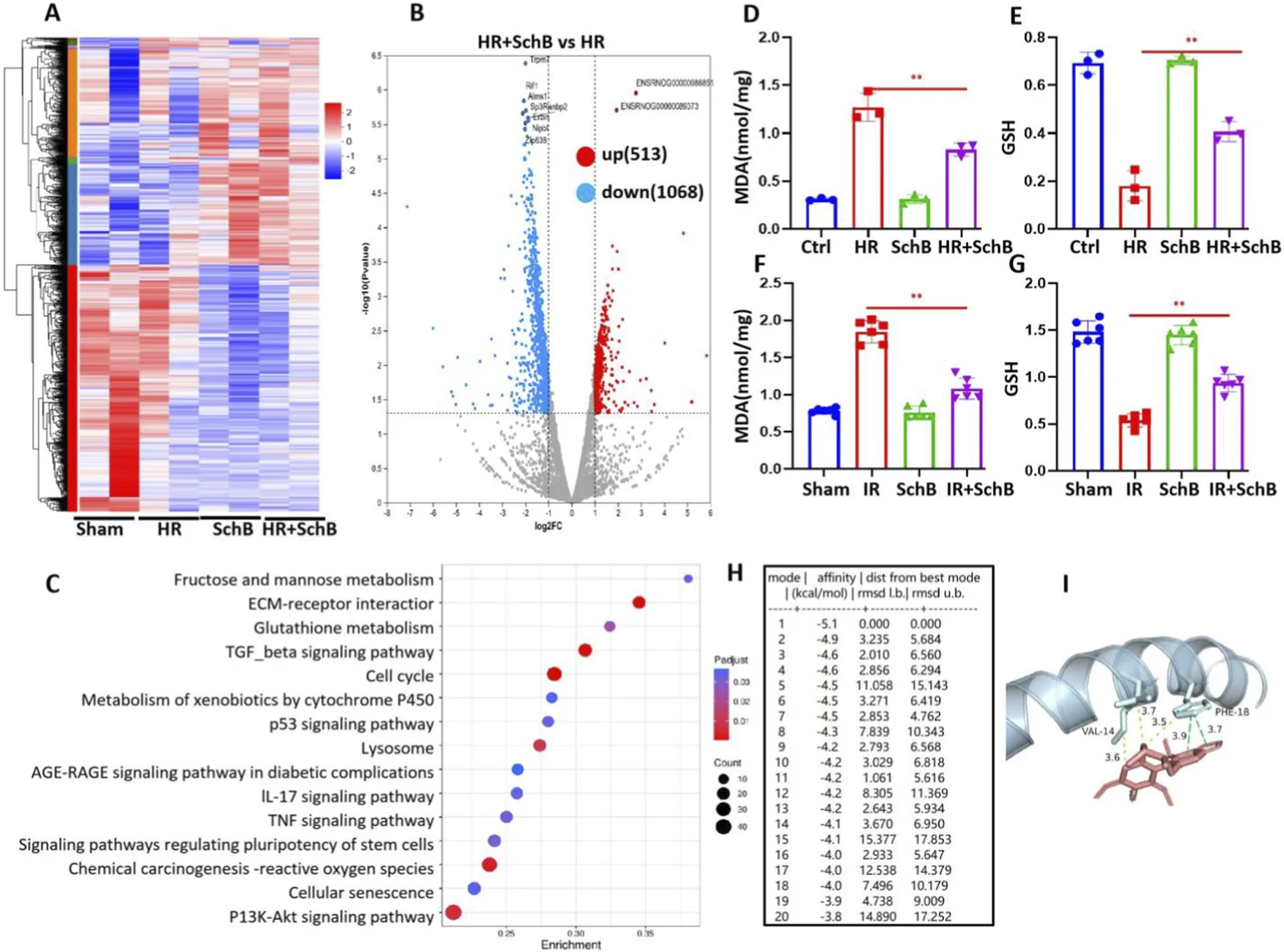
The renal protective effect of SchB stems from its inhibitory action on oxidative stress. **(A)** RNA sequencing of H/R-challenged RTECs treated with or without SchB. **(B)** Identification of 1,581 differentially expressed genes (DEGs): 513 upregulated and 1,068 downregulated by SchB, highlighting the extensive regulatory role of SchB in renal repair. **(C)** KEGG pathway enrichment analysis. **(D–G)** SchB reduced malondialdehyde (MDA) levels and enhanced GSH content in AKI. **(H,I)** Molecular docking of SchB to the CTR1 copper-binding pocket (binding energy −5.1 kcal/mol), forming hydrophobic interactions with Val14 and π–π stacking with Phe18. Data are expressed as mean ± SD from a minimum of three independent replicates per experiment (n = 3 per group). Comparisons between two groups were performed using an unpaired Student’s t-test. **P < 0.01 vs. indicated groups. Statistical significance was defined as P < 0.05.

#### 3.2.4 SchB inhibits cuproptosis in a CTR1-dependent manner

Molecular docking was performed using AutoDock Vina to explore the potential interaction between SchB and CTR1. The top-ranked pose placed SchB in the predicted copper-binding pocket of CTR1, with a minimum binding energy of −5.1 kcal/mol, indicating a favorable binding affinity according to the stated threshold (<−5.0 kcal/mol for good binding activity). In this pose, SchB formed hydrophobic interactions with Val14 and π–π stacking interactions with Phe18 (Figures 8H,I), respectively. These findings suggest stable ligand–receptor engagement within the copper-coordinating region of the receptor.

SiRNA-mediated CTR1 knockdown (53% reduction, **P < 0.01) (Figures 9A,B) markedly attenuated SchB-mediated protection in H/R-challenged RTECs. In this CTR1-deficient, partially protected setting, SchB (25 μM, added at reoxygenation and maintained for 24 h) did not produce a statistically significant further reduction in any of measured parameter compared with si-CTR1 alone (*P* > 0.05; Figures 9C–L). This suggests that SchB provides no additional protection when CTR1-mediated copper entry is reduced.

**FIGURE 9:**
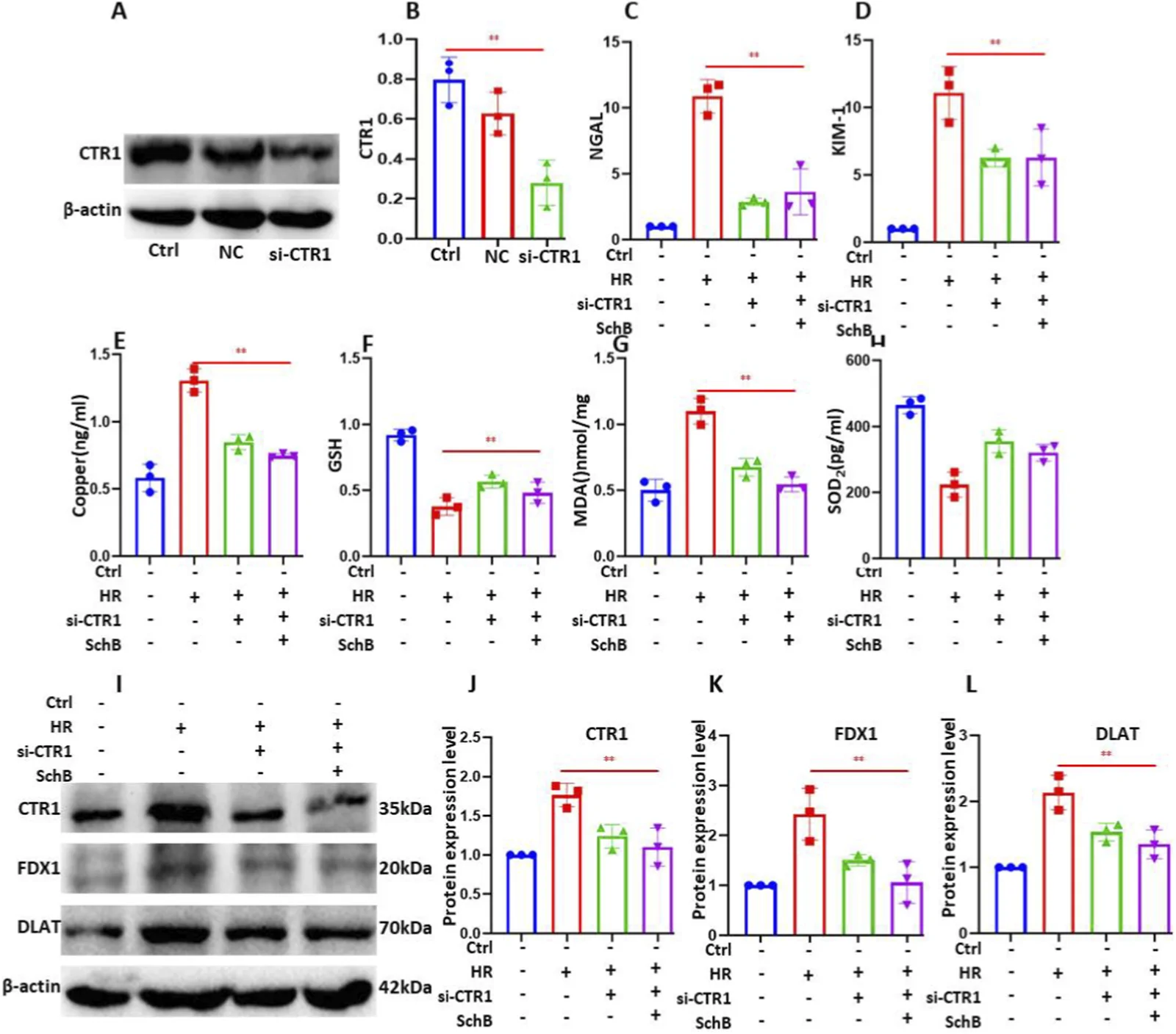
When CTR1 is silenced, the protective effect of SchB on HR-induced RTECs is significantly diminished **(A,B)**​ CTR1 expression in RTECs decreased by 53% following si-CTR1 administration. **(C–E)**​ When si-CTR1 was used in combination with SchB, no significant changes in renal tubular injury indicators or intracellular copper concentration were observed. **(F–H)**​ No significant differences were observed between the si-CTR1 and si-CTR1 + SchB groups for GSH, MDA, and SOD levels. **(I–L)**​ Cuproptosis-related proteins did not decrease significantly in the SchB + si-CTR1 group compared to the si-CTR1 group. Data are expressed as mean ± SD from a minimum of three independent replicates per experiment (n = 3 per group). Comparisons among multiple groups were performed using one-way ANOVA with Tukey’s *post hoc* test. **P < 0.01 vs. indicated groups. Statistical significance was defined as P < 0.05.

#### 3.2.5 SchB shows comparable or greater efficacy than TTM in attenuating cuproptosis and renal injury

To assess the therapeutic potential of SchB relative to a known copper chelator, we compared SchB (25 μM) and tetrathiomolybdate (TTM; 40 μM) in NRK-52 E cells subjected H/R. Compared with the H/R vehicle group, both treatments significantly reduced LDH release, KIM-1, and NGAL mRNA levels (Figures 10A–C), and intracellular copper content (Figure 10D; P < 0.01 for both treatments). SchB produced numerically greater reductions in KIM-1 (23%) and NGAL (18%) than TTM, although these between-treatment differences were not statistically significant.

**FIGURE 10:**
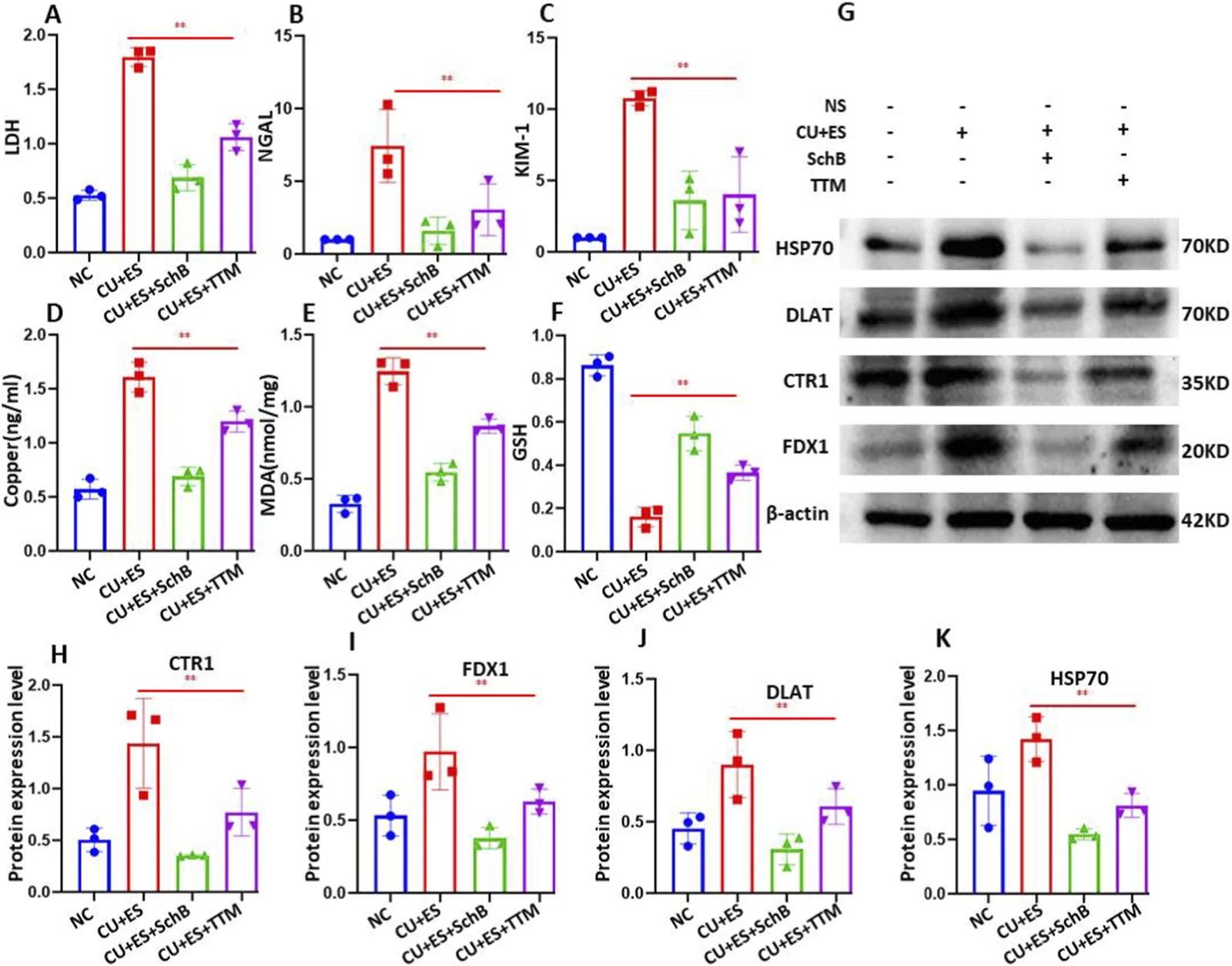
SchB exhibits comparable efficacy to TTM in attenuating cuproptosis and renal injury *in vitro*.​ **(A–C)** LDH release, NGAL mRNA, and KIM-1 mRNA levels. **(D)** Intracellular copper content. **(E,F)** Oxidative stress markers including MDA and GSH levels. **(G)** Representative Western blot images for cuproptosis-associated proteins and stress-responsive markers (DLAT, CTR1, FDX1,HSP70) with β-actin as the loading control. **(H–K)** Quantification of protein expression levels for CTR1, FDX1, DLAT, and HSP70 (n = 3 independent experiments). Direct comparisons between the SchB and TTM groups showed numerically greater reductions in KIM-1 and NGAL for SchB, but the differences did not consistently reach statistical significance. Both agents showed similar efficacy in reducing intracellular copper. Molecular profiling indicated that SchB downregulated FDX1, CTR1, DLAT, and HSP70 more effectively than TTM. These results support that SchB has efficacy at least comparable to that of TTM. Data are expressed as mean ± SD from a minimum of three independent replicates per experiment (n = 3 per group). Comparisons among multiple groups were performed using one-way ANOVA with Tukey’s *post hoc* test. **P < 0.01 vs. indicated groups. Statistical significance was defined as P < 0.05.

Both treatments attenuated oxidative stress, as shown by lower MDA and higher intracellular GSH levels (Figures 10E,F). Molecular profiling showed greater reductions in the cuproptosis-associated proteins FDX1, CTR1, and DLAT and the stress-responsive marker HSP70 with SchB than with TTM (Figures 10G–K).

Together, these findings suggest that SchB has effects comparable to those of TTM in this *in vitro* H/R model. Although SchB showed numerically greater effects on some endpoints, the differences between the treatments were modest and did not consistently reach statistical significance.

## 4 Discussion

Our findings highlight three aspects of AKI biology that have potential therapeutic implications. First, pathway gene set scoring assigned approximately one-third of RTECs to cuproptosis, making it the most frequently assigned regulated RTEC death modality. Consistent with the role of this pathway in human AKI, clinical biopsy samples showed increased CTR1 and FDX1 expression in tubular epithelial cells across a cohort comprising predominantly nephrotoxic, toxin-related, and infection-related cases (Table 1). Although the cohort included only one case of isolated ischemic AKI, these findings suggest that cuproptosis may contribute to tubular injury across multiple AKI etiologies. Second, SchB’s protective effect appears to depend, at least in part, on CTR1, and SchB showed effects comparable to those of the copper chelator tetrathiomolybdate (TTM) under *in vitro* conditions. Third, these findings provide a preclinical rationale for strategies aimed at preserving physiological copper function and limiting pathological copper accumulation.

RTEC injury is a central pathophysiological event that drives the onset and progression (Zhong et al., 2025; Zhou et al., 2025). While necrosis, apoptosis, pyroptosis, autophagy, and ferroptosis have been extensively implicated in tubular cell demise (Wang J. et al., 2024; Li X. et al., 2024; Yuan et al., 2024; Wei et al., 2025), cuproptosis represents a recently identified RCD modality triggered by copper concentrations exceeding cytotoxic thresholds (Jiang et al., 2023; Yang et al., 2024). Unlike apoptosis, necrosis, and ferroptosis, copper-dependent cell death involves the direct copper-mediated modification of lipoylated TCA cycle proteins, causing protein aggregation, iron–sulfur cluster instability, and proteotoxic stress (Tsui et al., 2024). Our single-cell transcriptomic analysis, together with multi-model validation across human tissues, murine I/R kidneys, and H/R-challenged RTECs, aligns with emerging evidence linking copper dyshomeostasis to kidney disease (Teschke and Eickhoff, 2024), suggesting that ischemia disrupts both CTR1-mediated import and ATP7B-dependent export. The specific enrichment of cuproptosis in proximal tubules, which are metabolically active cells dependent on mitochondrial tricarboxylic acid cycling, supports the mechanistic link between copper overload and lipoylated enzyme aggregation (Tsvetkov et al., 2022). Furthermore, the strong positive correlations between CTR1/FDX1 expression and established injury biomarkers (SCr, BUN, and urinary NAG], as well as the inverse correlation with eGFR, suggest the translational potential of cuproptosis markers in AKI diagnosis and risk stratification.

A limitation of our single-cell gene-set scoring approach is its inability to distinguish baseline metabolic gene expression from active cell death program activation. Proximal tubular epithelial cells constitutively express high levels of TCA cycle and lipoylation genes due to their mitochondrial density and reliance on oxidative phosphorylation. Consequently, the assignment of cuproptosis as the top-ranked RCD modality in these cells may partially reflect intrinsic metabolic features rather than specific upregulation of cuproptosis signaling. Future studies employing orthogonal approaches—such as assessing DLAT oligomerization, iron-sulfur cluster protein stability, and FDX1-dependent functional rescue assays—are needed to differentiate cuproptosis execution from a state of copper susceptibility.

Natural products have played a sustained role in drug discovery and continue to contribute substantially to the development of new medicines (Newman and Cragg, 2020; Zhang et al., 2024; Liu L. et al., 2026; Miao and Vaziri, 2025; Liu et al., 2026b; Lin et al., 2024). Increasing evidence suggests that multiple natural compounds ameliorate renal injury through diverse molecular mechanisms, including antioxidative and anti-inflammatory effects (Li et al., 2024c; Gao et al., 2025; Wang Y. N. et al., 2024; Ben-Azu et al., 2025; Liu et al., 2026c). SchB has attracted considerable attention because of its antioxidant properties and mechanisms of action (Fang et al., 2025; Wu J. et al., 2024). As mitochondria are major targets of oxidative stress, they may particularly benefit from the ability of SchB to preserve both structural and functional integrity through multiple mechanisms (Chen et al., 2024; Hong et al., 2023). The structural basis for the function of SchB as a CTR1 modulator may be attributed to its hydrophobic dibenzocyclooctadiene scaffold, which exhibits favorable complementarity for insertion into the extracellular domain pocket of CTR1. This molecular interaction may attenuate the toxic effects of heavy metals on cellular structure and function (Nasser et al., 2020). Molecular docking studies revealed that SchB exhibited moderate binding affinity for CTR1. Subsequent experiments showed that the inhibition of CTR1 followed by SchB treatment failed to produce additional protective effects, suggesting that SchB exerts renoprotective effects requiring CTR1 regulation. We acknowledge that only ATP7B was assessed in the present study, whereas ATP7A, a major copper efflux transporter in most epithelia, was not examined. Future studies investigating both transporters would provide a more complete understanding of copper clearance impairment in ischemic AKI.

RNA sequencing analysis showed that the SchB + H/R group exhibited 513 upregulated and 1,068 downregulated genes compared to the H/R group, indicating the extensive regulatory role of SchB in AKI repair. KEGG analysis demonstrated that SchB-regulated DEGs were significantly enriched in glutathione metabolism, p53 signaling, cellular senescence, PI3K-Akt pathways. As cuproptosis is largely independent of reactive oxygen species, the observed changes in GSH, MDA, and SOD2 do not necessarily indicate that SchB acts through a classical antioxidant mechanism. Instead, they may reflect reduced copper accumulation and cellular stress following CTR1 modulation. Thus, SchB may primarily act by altering upstream copper handling. Similar protective effects have been reported in other tissues, including the brain and myocardium (Hong et al., 2023; Tang et al., 2022). Molecular docking demonstrated that SchB exhibits moderate binding affinity for CTR1, and subsequent si-CTR1 experiments showed that SchB failed to provide additional protection or further reduce intracellular copper levels after CTR1 knockdown. These results suggest that SchB exerts AKI-protective effects via CTR1.

This study demonstrated that cuproptosis is a predominant RCD modality underlying tubular epithelial cell injury in I/R-induced AKI (Zou et al., 2024; Lv and Yu, 2025). To our knowledge, this is one of the first studies to link cuproptosis to ischemic AKI and identify CTR1 as a potential drug target. The mechanism of action of SchB differs substantially from that of the classical copper chelator TTM. TTM may disrupt physiological copper functions through non-selective chelation, whereas SchB appears to modulate copper uptake through CTR1. Under the tested conditions, SchB provided protection to tubular epithelial cells comparable to that of TTM, with numerically greater effects on some endpoints.

In summary, we identified cuproptosis as a druggable form of RCD in AKI and characterized SchB as a CTR1-dependent therapeutic candidate. By modulating copper entry rather than indiscriminately chelating systemic copper, SchB represents a mechanistically distinct and clinically promising strategy for mitigating ischemic tubular injury, providing a strong preclinical rationale for repurposing this natural product in acute ischemic kidney disease. Although SchB is known to have antioxidant properties, the effects observed here may reflect improved cellular homeostasis following a reduction in the copper burden, rather than the direct reversal of the core cuproptotic cascade. Mechanistically, SchB may protect against cuproptosis through two mechanisms: blocking copper influx requiring CTR1 or reducing CTR1 protein levels via transcriptional or post-transcriptional regulation. Although our current findings establish the functional dependency of SchB-mediated protection on CTR1, the precise molecular mechanism underlying SchB-induced CTR1 downregulation, whether through enhanced protein degradation, transcriptional suppression, or post-transcriptional regulation, remains to be elucidated. Addressing these possibilities will require further investigation, including analyses of CTR1 mRNA stability, ubiquitination status, and subcellular trafficking dynamics. Elucidating the precise molecular basis of SchB-mediated CTR1 modulation represents an important direction for future investigations and may provide deeper insights into the dual role of SchB as a functional modulator and regulator of CTR1 expression. We acknowledge that DLAT aggregation and disruption of the protein lipoylation axis are more definitive cellular indicators of cuproptosis than total DLAT abundance measured by western blotting. In this study, total DLAT and HSP70 levels were considered supportive biochemical findings alongside FDX1 and CTR1, rather than standalone evidence of cuproptotic execution.

Although this study has several limitations, its preliminary findings offer mechanistic insights. First, the clinical biopsy cohort was small and drawn from a single center; therefore, multicenter validation across diverse AKI etiologies is needed. Second, a direct comparison of SchB and TTM potency is complicated by their distinct pharmacokinetic properties and the use of single, literature-based effective concentrations without full dose–response curves. Accordingly, we cannot draw firm conclusions about whether SchB offers a renoprotective advantage over TTM. Third, the Cu/ES experimental model involves inhibition of the mitochondrial electron transport chain rather than true hypoxia. Cuproptosis-related changes should therefore be interpreted alongside findings from the *in vivo* I/R model. Fourth, the CTR1 knockdown experiment warrants cautious interpretation. The absence of further improvement with SchB in si-CTR1 cells suggests that its protective effect may require a CTR1-competent state. However, this finding alone does not establish direct physical binding between SchB and CTR1 or show that CTR1 is SchB’s exclusive target. Finally, the absence of an H/R + negative control siRNA + SchB group in the same experimental batch limits cross-group comparisons. Future studies using CTR1 overexpression or rescue experiments, CETSA, DARTS, or co-crystallization approaches are needed to establish direct target engagement.

## 5 Conclusion

Cuproptosis was identified as a druggable target in AKI, and SchB was characterized as a novel CTR1-targeted therapeutic agent for AKI. These findings provide a preclinical rationale for the repurposing of this natural product for the treatment of ischemic kidney disease.

## Data Availability

The raw data supporting the conclusions of this article will be made available by the authors, without undue reservation.
